# Aortic dissection associated with transcatheter aortic valve replacement: Implications of preexisting aortic pathologies

**DOI:** 10.1016/j.xjse.2025.100051

**Published:** 2025-04-09

**Authors:** Tyler M. Bauer, Himanshu J. Patel, David Williams, Stanley J. Chetcuti, P. Michael Grossman, G. Michael Deeb, Shinichi Fukuhara

**Affiliations:** a Department of Cardiac Surgery, University of Michigan, Ann Arbor, Mich; b Department of Radiology, University of Michigan, Ann Arbor, Mich; c Division of Cardiovascular Medicine, University of Michigan, Ann Arbor, Mich

**Keywords:** transcatheter aortic valve replacement, type A aortic dissection, type B aortic dissection, thoracic endovascular aortic repair, ascending aortic aneurysm

## Abstract

**Objectives:**

Acute aortic dissection is a rare yet life-threatening complication associated with transcatheter aortic valve replacement (TAVR). Given its low incidence, the characteristics and risk factors remain inadequately investigated.

**Methods:**

Between 2011 and 2023, a total of 2558 TAVR procedures were performed at the University of Michigan. Among these, 12 patients (0.47%) developed aortic dissection. Additionally, 3 post-TAVR patients from other institutions presented with aortic dissection and were treated at our institution, yielding a total of 12 type A and 3 type B aortic dissections.

**Results:**

The median age was 79.5 years, and 5 (33.3%) exhibited end-organ malperfusion affecting the brain (n = 3), legs (n = 3), and kidneys (n = 1). TAVR device migration requiring repositioning was observed in 40% (6 out of 15) of cases. In type A dissections, the entry tear consistently occurred along the greater curvature. Patients with type A dissections had a larger pre-TAVR aortic diameter than those without dissection (41.6 mm vs 34.5 mm; *P* < .001). Preexisting aortic dilation (≥45 mm) was associated with a significantly increased risk of type A dissection (odds ratio, 12.0; 95% confidence interval 3.0-50.6; *P* < .001). Type A dissection was managed with open repair in 7 patients (58.3%), all of whom survived 30 days, and with endovascular aortic repair in 4 patients (33.3%), all of whom experienced mortality; 1 patient received palliative care. All type B dissections were successfully treated with endovascular repair.

**Conclusions:**

TAVR-related aortic dissection is characterized by preexisting aortic risk factors with various mechanisms. Current surgical guidelines recommending aortic repair for dilatation ≥45 mm should be strongly considered in patients undergoing TAVR evaluation.

**Figure:**
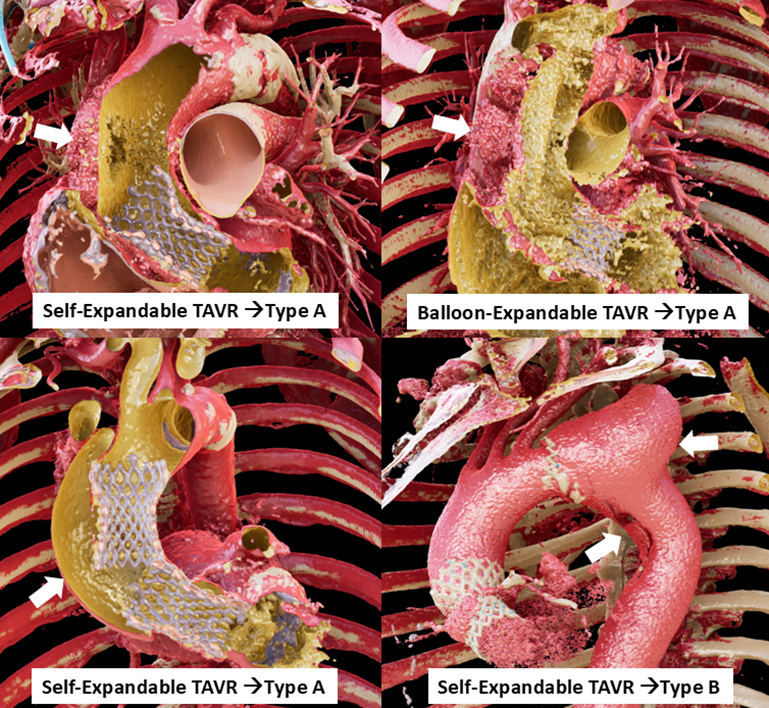
TAVR-associated aortic dissections with various clinical and anatomic characteristics.

**Central Message**

TAVR-associated aortic dissection is lethal, linked to preexisting risk factors. Prompt surgery is warranted for type A, whereas endovascular repair is effective for complicated type B dissections.

**Perspective**

TAVR-related aortic dissection is infrequent but highly lethal, occurring with various mechanisms and timings. Prompt surgical or endovascular management is warranted. Underlying aortic pathologies are highly prevalent in this subset. The current guideline recommendation of aortic dilatation ≥45 mm for concurrent aortic repair should be considered for patients undergoing TAVR workup.

Transcatheter aortic valve replacement (TAVR) has evolved from an investigational device to the predominant form of treatment for severe aortic stenosis in the United States.^1^ Although TAVR has displayed low procedural complications compared with surgical aortic valve replacement (SAVR), morbidity and mortality remain present.^2^

Aortic dissection is among the most lethal complications associated with TAVR.^3^ Acute aortic dissection, irrespective of type A or B, can occur at any time during the interaction between the guidewires/catheters, any component of the TAVR delivery system, or the TAVR valve frame and the aortic wall.^4^, ^5^, ^6^ The gold standard for managing type A dissection is surgical repair; however, off-label thoracic endovascular aortic repair (TEVAR) may be employed in selected high-risk cases with suitable anatomy. Acute type B aortic dissections may be managed either medically or endovascularly, contingent upon the location, extent of dissection, and presence of end-organ malperfusion.^7^ Current registry data estimate the incidence of aortic dissection associated with TAVR at 0.2% to 1.9%, without differentiation between type A or type B aortic dissections, nor does it report on the additional interventions used for management or provide detailed data to inform effective management strategies.^3^^,^^8^, ^9^, ^10^, ^11^, ^12^ Although the incidence remains exceedingly low, literature concerning case series or institutional-level experiences in managing this complication is scarce. The present study delineates the incidence, characteristics, management, and outcomes of aortic dissections following TAVR, based on more than a decade of institutional experience.

## Methods

The University of Michigan Institutional Review Board approved all aspects of the study (HUM00190884; approved August 6, 2020). The approval included a waiver of informed consent.

### Patient Selection

This study is retrospective and observational; 2558 TAVR cases, comprising 346 balloon-expandable, 2196 self-expandable, and 16 mechanically expandable valves, were performed between January 2011 to December 2023. All instances of aortic dissection were identified and classified as type A or type B aortic dissections. Additionally, patients who presented with aortic dissection after undergoing the procedure at outside institutions were included. Pre-TAVR computed tomography angiography (CTA) was reviewed to determine high-risk anatomic features and operative reports were analyzed alongside interviews with TAVR operators to elucidate the mechanism underlying aortic dissection. Follow-up clinical and radiographic data were complete, and no patients with aortic dissections after TAVR were lost to follow-up as of March 2025.

To determine the incidence of post-TAVR aortic dissections, review of all patients undergoing TAVR at our institution was conducted, with patients who have received a TAVR at outside institutions were excluded from this particular analysis. Clinical data for patients undergoing TAVR who did not develop aortic dissections were tabulated, including age and gender. TAVR CTA measurements were extracted from the clinical database, when available (2058 out of 2558 [80.3%]). Maximal thoracic aortic diameter and aortic angle were compared between patients who developed an aortic dissection, and those who did not. Additionally, thoracic aortic diameter was compared across an age- and gender-matched cohort (1 dissection case to 3 controls) to control for patient factors. The odds of developing a type A aortic dissection after TAVR in patients with a preexisting thoracic aortic dilatation (maximal diameter ≥45 mm) was tabulated. The 3-dimensional reconstruction of CTA image was performed using Viewtify (Sciement Inc).

### Statistical Analysis

Continuous variables are expressed as mean ± SD for normally distributed variables and medians with interquartile range for nonnormally distributed variables. Categorical variables are presented as proportions and absolute numbers. Differences among groups were detected using the χ^2^ test or Fisher exact test for categorical variables and Student *t* test or Mann-Whitney *U* test for continuous variables. All *P* values were the result of 2-tailed tests. The statistical analyses were performed using Stata version 14.2 (StataCorp).

## Results

**Figure 1:**
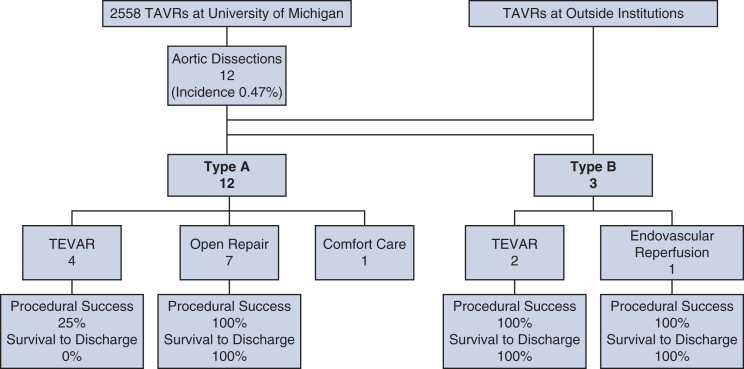
Study cohort. *TAVR*, Transcatheter aortic valve replacement; *TEVAR*, thoracic endovascular aortic repair.

A total of 15 patients (2 balloon-expandable and 13 self-expandable valves) with aortic dissection were treated at our institution. Of these, 12 patients received TAVR at our facility, consisting of 9 patients with type A aortic dissection and 3 patients with type B, with an overall incidence of 0.47% (12 out of 2558 TAVR implants). The incidence of any aortic dissection by TAVR valve type was 0.30% (out of 346 implants) for balloon-expandable valves, and 0.50% (out of 2196 implants) for self-expandable valves (*P* = .59). Furthermore, there was no difference in type A dissections by valve type (0.29% in balloon expandable vs 0.32% in self-expandable; *P* = .93). No patients received extreme TAVR oversizing (>30%). Additionally, 3 patients who experienced type A dissection post-TAVR at outside institution were transferred to our center for surgical intervention (Figure 1).

Baseline characteristics are shown in Table 1. Overall, patients were frail and had multiple comorbidities. The median age and sex distribution were similar between patients who developed dissections compared with those who did not (age, 79.0 vs 79.0 years; male sex, 60.0% vs 56.3%).

**Table 1:** Patient demographics (N = 15)

Characteristic	Result
Age (y)	79 (72-86)
Female	6 (40.0)
Diabetes	3 (21.4)
Frailty	9 (60.0)
Previous stroke	3 (20.0)
Coronary artery disease	7 (45.6)
Chronic kidney disease	6 (40.0)
Dialysis	1 (6.7)
Home oxygen therapy	3 (20.0)
Genetically triggered aortic disease	0
STS-PROM (%)	8.5 (7.3-14.3)
Previous cardiovascular surgery	
SAVR	2 (13.3)
Aortic root repair	2 (13.3)
Ascending and partial aortic arch repair	2 (13.3)

Values are presented as n (%) or median (interquartile range). *STS-PROM*, Society of Thoracic Surgeons Predicted Risk of Mortality; *SAVR*, surgical aortic valve replacement.

### Type A Aortic Dissections: Timing and Mechanisms

Aortic characteristics, management, and clinical outcomes are summarized in Table 2. Of the 12 patients experiencing type A aortic dissections, consisting of 8 DeBakey type 1 and 4 DeBakey type 2, all had an entry tear along the greater curvature of the aorta, with the sinotubular junction as the most common site. Six patients experienced type A aortic dissection during the TAVR procedure, with 2 cases occurring in the preimplant phase, presumably due to catheters, TAVR delivery system passage, and/or preimplant balloon valvuloplasty. Of the patients with pre-TAVR implant dissections, 1 diagnosis was made via predeployment aortography, and the other by transesophageal echocardiography. Of the 4 patients who developed type A dissection postdevice deployment, 1 was complicated by annular rupture following a self-expanding TAVR. Two of these postdeployment dissections were likely related to device migration/repositioning using snares, whereas the others were attributed to device passage in the presence of underlying aortic aneurysms. In contrast, 4 of 12 patients had type A dissection postdischarge (2 days to 7 years); all were likely due to mechanical stent frame erosion into the aorta in the presence of underlying aortic aneurysm. Lastly, 2 patients were diagnosed with focal type A dissection during evaluation for AV reintervention.

**Table 2:** Aortic characteristics and clinical outcomes associated with type A aortic dissections after transcatheter aortic valve replacement (TAVR)

No.	Age, sex	Valve type, size (mm)	Aortic risk factors	Diagnosis timing (imaging)	TAVR migration	Intimal tear location	Aortic dissection mechanism	Associated complications	Management	Procedural success	Outcome
1	86, M	BEV, 29	- Dilated ascending aorta (46 mm) - Dilated distal arch (52 mm) - Horizontal aorta (63°)	Post-deployment (aortography, dampened A-line)	No	STJ, greater curvature	TAVR deployment	Cerebral malperfusion	TEVAR	No (incomplete tear coverage)	Death (POD 2)
2	79, F	SEV, 26	- Dilated ascending aorta (43 mm) - Horizontal aorta (67°) - Abdominal aortic aneurysm (64 mm)	Post-deployment (aortography, dampened A-line)	No	Mid ascending aorta, greater curvature	Delivery system passage	Cerebral malperfusion and hemopericardium	TEVAR + innominate artery stenting	No (incomplete tear coverage)	Death (POD 5)
3	71, F	SEV, 29	- Dilated ascending aorta (45 mm)	Pre-deployment (aortography)	No	STJ, greater curvature	Delivery system passage	None	TEVAR	Yes	Death (POD 18)
4	93, F	SEV, 34	- Dilated ascending aorta (45 mm) - Horizontal aorta (69°)	1.5 mo post-TAVR (CTA)	No	Mid ascending aorta, greater curvature	Stent frame erosion	None	TEVAR	No (incomplete tear coverage)	Death (POD 5)
5	72, M	SEV, 29	- Dilated ascending aorta (43 mm)	2 y post-TAVR (CTA)	No	STJ, greater curvature	Stent frame erosion	Large pseudoaneurysm	Root and ascending repair	Yes	Alive (3 y)
6	80, F	BEV, 26	- Dilated ascending aorta (48 mm) - Bicuspid valve	2 y post-redo-TAVR (CTA)	Yes (retrograde)	STJ, greater curvature	Stent frame erosion	Cerebral malperfusion	Ascending + hemiarch repair	Yes	Alive (3 y)
7	71, M	SEV, 29	- Bicuspid valve - Horizontal aorta (58°) - Previous aortic repair with graft kink - Dilated native STJ/root (42 mm)	0.5 mo post-TAVR	Yes (antegrade, repeat recapturing)	Non-coronary sinus of the aortic root	Delivery system passage	None	Root repair	Yes	Alive (2 y)
8	32, M	SEV, 26	- Autograft aneurysm (47 mm) - 3 previous heart surgeries	7 y post-TAVR (CTA)	No	Autograft STJ, greater curvature	Stent frame erosion	None	Root repair	Yes	Alive (2 y)
9	86, M	SEV, 29	- Dilated ascending aorta (54 mm) - Horizontal aorta (71°) - Aortic tortuosity	Pre-deployment (dampened A-line, aortography)	Yes (antegrade, repeat recapturing)	STJ, greater curvature	Delivery system passage	Renal and leg malperfusion	Comfort care	N/A	Palliative care
10	86, F	SEV, 29	- Horizontal aorta (56°)	2 d post-TAVR (CTA)	Yes (antegrade, second valve)	Greater curvature of ascending aorta	TAVR deployment and recapture	None	Ascending + hemiarch repair	Yes	Alive (3 y)
11	78, M	SEV, 34	- Dilated aorta (45 mm) - Bicuspid valve	Post-deployment (TTE)	No	STJ, (greater curvature)	Balloon valvuloplasty for PVL and TAVR under-expansion	Root rupture and hemopericaridum	Root + ascending repair	Yes	Death (6 mo)
12	76, M	SEV, 34	- Horizontal aorta (58°) - Abdominal aortic aneurysm	Post-deployment (TEE)	Yes (antegrade, repeat recapturing)	STJ, greater curvature	TAVR deployment and recapture	None	Root + ascending repair	Yes	Alive (8 mo)

*M*, Male; *BEV*, balloon-expandable valve; *STJ*, sinotubular junction; *TEVAR*, thoracic endovascular aortic repair; *POD*, postoperative day; *F*, female; *SEV*, self-expandable valve; *CTA*, computed tomography angiography; *N/A*, not available; *TTE*, transthoracic echocardiography; *TEE*, transesophageal echocardiography.

Nearly all patients with post-TAVR type A dissection exhibited high-risk aortic features, including increased thoracic aortic diameter, tortuous aorta, abdominal aortic aneurysms, and/or bicuspid AV (Table E1). Additionally, TAVR device migration, often associated with horizontal aorta necessitating snaring, repositioning, and/or a second valve implantation was observed in 41.2% (5 out of 12) of cases.

### Type A Aortic Dissections: Management and Outcomes

**Figure 2:**
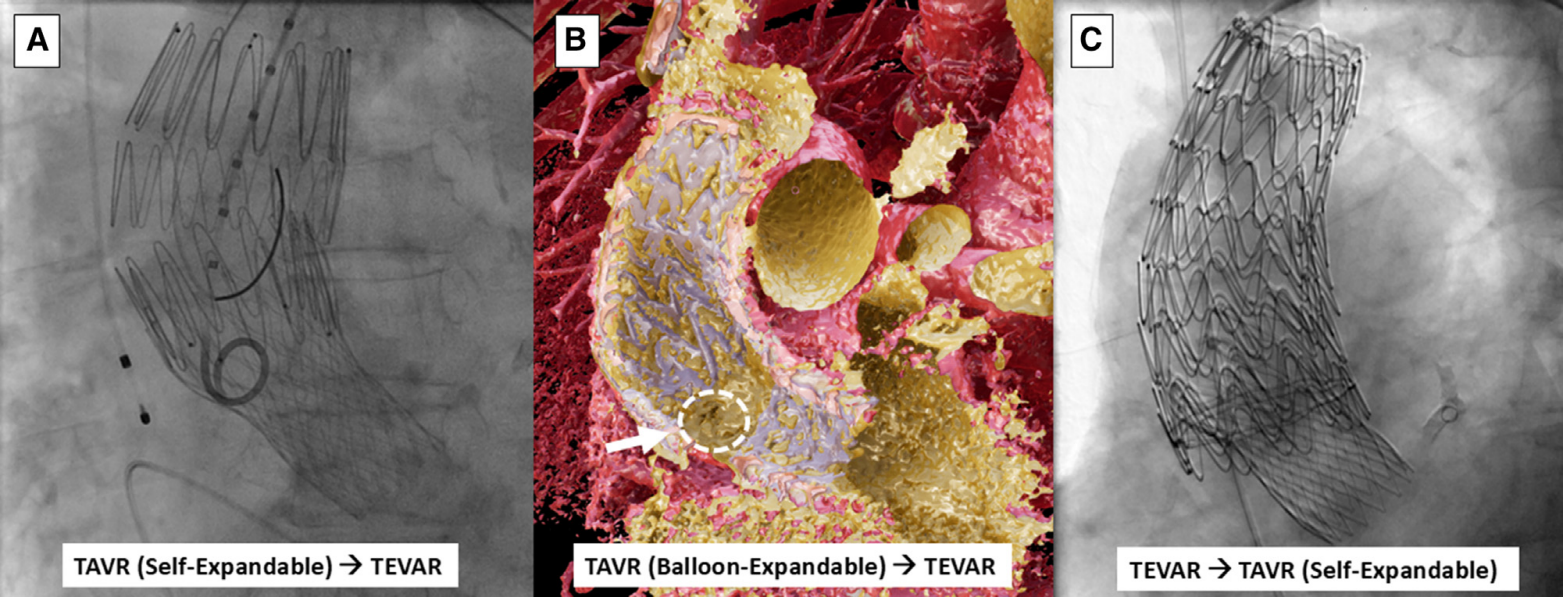
Thoracic endovascular aortic repair (*TEVAR*) to type A dissection after transcatheter aortic valve replacement (*TAVR*). A, TAVR followed by TEVAR (TEVAR-in-TAVR configuration) in the setting of self-expandable valve. Interaction between the TEVAR and TAVR stent frame resulted in failure of entry tear coverage. B, TEVAR-in-TAVR in the setting of a balloon-expandable valve. *White arrow* and *dashed circle* represent uncovered intimal tear at the sinotubular junction. C, TEVAR followed by TAVR (TAVR-in-TEVAR configuration) in the setting of self-expandable valve. The intimal tear was completely covered by the stent-graft.

End-organ malperfusion was seen frequently, affecting the brain (n = 3), legs (n = 1), and kidneys (n = 1). Hemopericardium was seen in 2 patients. Four patients received a TEVAR as the primary intervention. If TEVAR was attempted after TAVR deployment, the aortic stent-graft was landed at the distal portion of the TAVR (TEVAR-in-TAVR approach, 3 patients) (Figure 2, *A* and *B*). In all cases, the TEVAR-in-TAVR configuration failed to cover the entry tear due to interaction between aortic stent-graft and TAVR valve, regardless of short- (Figure 2, *B*) or tall-frame TAVR valve (Figure 2, *A*), resulting in in-hospital mortality. Conversely, when TEVAR was performed first for type A aortic dissection identified during the pre-TAVR implant phase (TAVR-in-TEVAR, 1 patient) (Figure 2, *C*), the entry tear was successfully sealed by the aortic stent-graft. However, the patient, being frail at baseline, was transitioned to palliative care postoperatively. Seven patients underwent open surgical repair, typically comprising SAVR and ascending/hemiarch aortic repair, with all surviving to 30 days following surgery. Median intensive care unit and hospital length of stay was 7 days (range, 2-52 days) and 16 days (range, 7-73 days), respectively. All but 1 patient following open repair was alive 1 year following repair.

### Type A Aortic Dissections: Incidence and Association With Enlarged Thoracic Aortic Diameter

**Figure 3:**
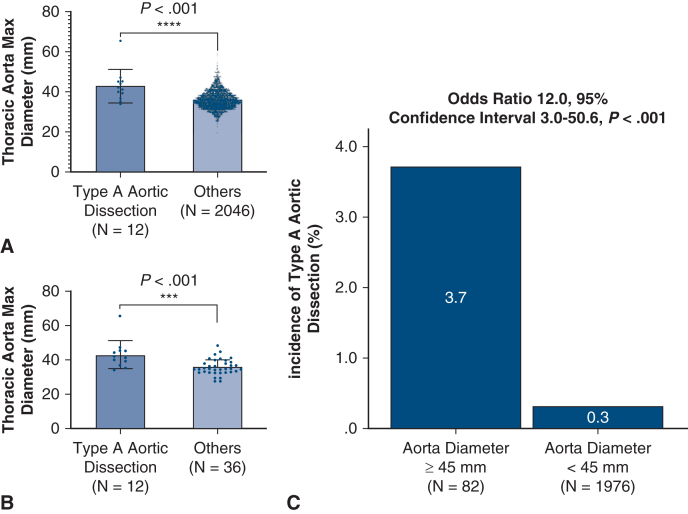
Relationship between baseline aortic diameter and occurrence of type A aortic dissection. Comparison of max thoracic aortic diameter in patients with type A aortic dissection after transcatheter aortic valve replacement (*TAVR*) (n = 12) compared with all other TAVR recipients (n = 2046) (A) and age- and gender-matched controls (n = 36) (B). C, The incidence of type A aortic dissections in patients with maximum aortic diameter ≥45 mm (n = 82) versus patients with <45 mm aortic diameter (n = 1976).

Among all TAVR patients during the study period, pre-TAVR CTA measurements were available for 80.4% (2058 out of 2558). The median thoracic aortic diameter in patients with type A dissection post-TAVR was 41.6 mm, compared with 35.4 mm in those without dissection (*P* < .001) (Figure 3, *A*). This finding was confirmed in an age- and gender-matched cohort (1 dissection case to 3 controls; 41.6 vs 34.5 mm; *P* < .001) (Figure 3, *B*).

The prevalence of a thoracic aortic dilatation, defined as maximum aortic diameter ≥45 mm, was 4.0% (82 out of 2058) on pre-TAVR CTA. The incidence of type A aortic dissections was 3.7% (3 out of 82) in those with preexisting aortic dilatation versus 0.3% (6 out of 1976) in those without (odds ratio, 12.0; 95% CI, 3.0-50.6; *P* < .001) (Figure 3, *C*). There was no significant difference in aortic angle between patients with and without type A aortic dissections (46.3° vs 45.5°).

### Type B Aortic Dissections

**Figure 4:**
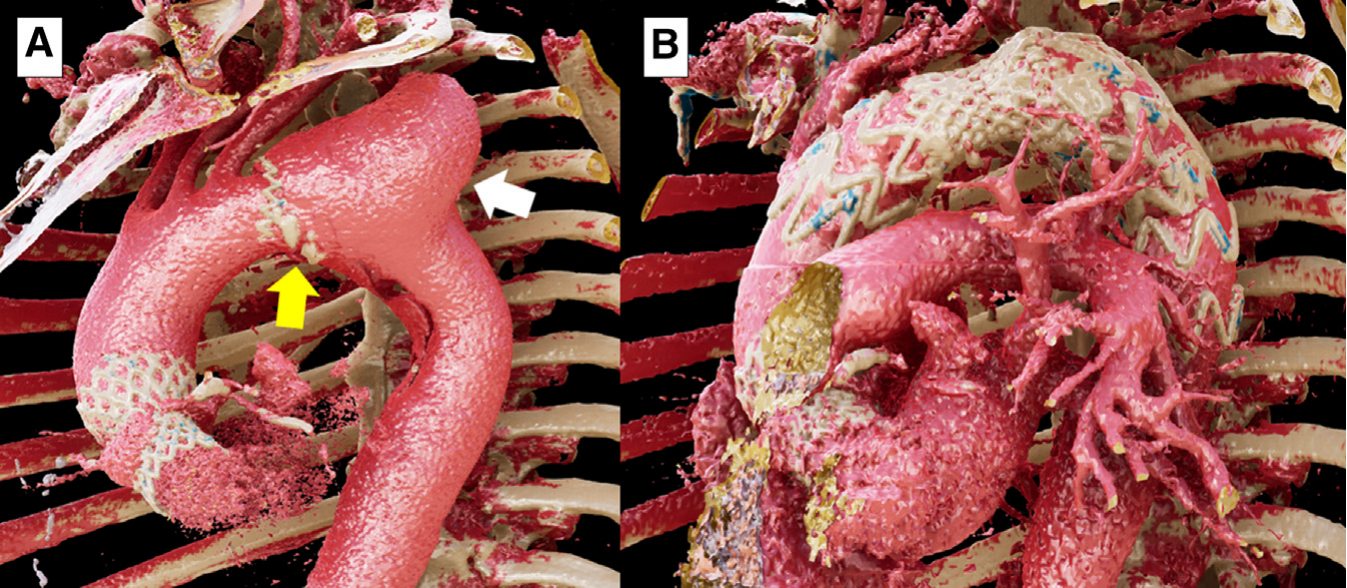
Thoracic endovascular aortic repair (*TEVAR*) to type B aortic dissection with distal arch aneurysm after transcatheter aortic valve replacement (*TAVR*). A, Preoperative computed tomography angiography (*CTA*) with 3-dimensional reconstruction. *White arrow* indicates the rapidly growing aortic aneurysm. *Yellow arrow* indicates an embolized valve located at the distal arch. B, Post-TEVAR CTA with complete exclusion of the aortic aneurysm by the stent-graft through the migrated TAVR valve.

All patients demonstrated high-risk aortic features, including dilatated aorta and gothic aortic arch with steep arch angulations (Table 3). All were identified during the TAVR procedure, with 1 readmitted 3 months later with back pain, and a rapidly enlarging distal arch aneurysm (Figure 4, *A*). This patient had an intimal tear from an embolized TAVR valve, which was as a result of snaring and repositioning distal to the left subclavian artery before implanting a second TAVR valve. Combining type A aortic dissection cases, TAVR device migration requiring snaring and repositioning was observed in 40.0% (6 out of 15) of all dissection cases. All type B dissections were managed with endovascular interventions: TEVAR (2 patients) (Figure 4, *B*) or endovascular reperfusion (1 patient). Endovascular reperfusion included supraceliac aortic septal fenestration to restore the true lumen perfusion. These procedures had a 100% success rate, with all patients surviving to discharge. Notably, the patient who underwent endovascular reperfusion without TEVAR developed a progressively enlarging aortic aneurysm >6 cm on follow-up and later died, likely due to aortic rupture, 7 years post-TAVR.

**Table 3:** Aortic characteristics and clinical outcomes associated with type B aortic dissections after transcatheter aortic valve replacement (TAVR)

No.	Age, sex	Valve type, size (mm)	Aortic risk factors	Diagnosis timing (imaging)	TAVR migration	Intimal tear location	Aortic dissection mechanism	Associated complications	Management	Procedural success	Outcome
13	80, M	SEV, 29	- Dilated ascending aorta (43 mm)	Post-deployment (aortography, dampened A-line)	Yes (antegrade, second valve)	Zone 3	TAVR deployment	Leg malperfusion and arch aneurysm	TEVAR (3 mo post-TAVR)	Yes	Alive (3 y)
14	92, F	SEV, 29	- Gothic arch with steep angulation	Post-deployment (TEE)	No	Zone 3	Delivery system passage	Retrograde type A dissection	TEVAR	Yes	Alive (7 y)
15	73, M	SEV, 31	- Gothic arch with steep angulation and aortic elongation - Previous ascending repair	Post-deployment (lack of femoral pulse)	No	Zone 3	Delivery system passage	Leg malperfusion	Endovascular reperfusion/fenestration	Yes	Death (8 y)

*M*, Male; *SEV*, self-expandable valve; *TEVAR*, thoracic endovascular aortic repair; *F*, female; *TEE*, transesophageal echocardiography.

## Discussion

This study presents among the largest reported cohorts of aortic dissections associated with TAVR in the literature. Uniquely, it provides data on TAVR procedural volume as a denominator, alongside incidence rates for both type A and type B dissections, stratified by implanted TAVR valve type. Over a 10-year period at a single institution, it is evident that although aortic dissection post-TAVR is rare, it entails substantial morbidity and mortality risks. This study identifies a 0.47% incidence of aortic dissections following TAVR, with type A dissections occurring nearly twice as frequently as type B. Almost all patients experiencing aortic dissection post-TAVR had underlying aortic pathology, with a larger thoracic aortic diameter being significantly associated with type A dissections. Additionally, TAVR device migration during implantation was frequently observed in this cohort, likely due to an underlying dilated aorta, posing challenges in securing TAVR. Open surgical approaches for type A dissections and endovascular strategies for type B dissections appear effective, although patients treated with TEVAR for type A dissections did not survive to discharge.

Thoracic aortic dilation commonly coexists with aortic stenosis, influencing approximately 20% of patients undergoing AVR, especially those with bicuspid valve disease.^10^ Despite the high prevalence of aortic dilation, the current American College of Cardiology/American Heart Association and European Society of Cardiology guidelines lack specific management recommendations for TAVR candidates,^12^^,^^13^ contrasting with their clear guidance on concurrent aortic repair during SAVR when the aortic diameter reaches 4.5 cm or greater. In this context, many centers prioritize a multidisciplinary approach and personalized interventions over a standardized threshold for managing thoracic aortic aneurysms for TAVR candidates.

Fan and colleagues^8^ examined outcomes in 111 patients with bicuspid valves who received a self-expandable TAVR, grouping them by aortic dilation presence. The incidence of type A dissection was 1.8%. Using a conservative threshold for dilation (>40 mm), they observed increased all-cause mortality in patients with dilated aortas. Jia and colleagues^10^ reported on 261 TAVR patients, 91 of whom had bicuspid valves and ascending aortic dimensions ≥40 mm, finding a 4.4-fold higher 4-year mortality in those with larger diameters. Ochiai and colleagues^11^ assessed 1426 patients with tricuspid aortic stenosis, finding higher rates of paravalvular regurgitation and secondary valve implantation in the dilated aorta group, along with significantly elevated 2-year all-cause mortality. Collectively, these findings raise concerns about TAVR use in patients with thoracic aortic dilation.

In this study, combined type A and type B dissection incidence was 0.47%, consistent with available data in the literature. Seven cases required same-day emergency operations after TAVR, which represented 41.1% (7 out of 17) of all same-day TAVR-related surgical conversions during the study period. No difference in dissection occurrence was noted between balloon-expandable and self-expandable TAVRs, both for combined type A and B dissections, and for type A dissections alone. Unlike previous studies, this investigation differentiates dissection location, reporting nearly a 2:1 incidence of type A to type B dissections post-TAVR. Previous investigations have not identified risk factors for aortic dissection after TAVR. Our cohort of dissection cases resembled the nondissection group in age and gender, aligning with prior studies.^8^, ^9^, ^10^, ^11^ Notably, patients with aortic dissections frequently had preexisting aortic pathologies. Most significantly, the maximum thoracic aortic diameter in type A dissection cases was, on average, >5 mm larger than in nondissection cases. Although rare, the risk of type A dissection was approximately 12-fold higher in patients with aortic diameters exceeding 45 mm. This elevated risk in the context of aortic dilatation warrants consideration during shared decision making for TAVR versus SAVR. TAVR device migration necessitating snaring or secondary implantation occurred in 40% of cases in this study, underscoring the TAVR procedural risks along with higher incidence of paravalvular regurgitation posed by baseline aortic dilation and tortuosity, as previously reported.^11^ The mechanism by which valve migration leads to higher rates of aortic dissections was indeed clear. The combination of dilated aorta (tissue friability or risk for TAVR migration), horizontal aorta (risk for TAVR migration), and valve manipulation/repositioning (risk for direct aortic trauma) can lead to the vicious cycle of aortic dissection occurrence.

For managing these complications, traditional approaches remain appropriate, with surgical repair as the standard for type A dissections and endovascular or nonoperative management for type B dissections, even in the presence of TAVR. Notably, all type A entry tears occurred between the aortic root and mid ascending aorta along the greater curvature. These aortic dissections still occurred within the native aorta remnant in patients with previous aortic repair (patients 7, 8, and 15). In our study, 33% of type A dissections were managed with off-label TEVAR due to high-risk patient profiles. Ascending TEVAR using the dedicated ASG device (W.L. Gore & Associates) in the ARISE study has demonstrated promising results^14^; however, it was challenging after TAVR given the TAVR valve's presence and limited landing zones near coronary or head vessels; the primary entry tear was only covered in a single case. Conversely, open repair was performed in 7 type A cases, all resulting in 30 days survival. Thus, for patients with aortic diameters ≥4.5 cm, SAVR with concurrent aortic repair should be considered for operable patients instead of TAVR.

### Study Limitations

Several study limitations warrant discussion. First, as a retrospective single-center study, our findings are associative and may lack generalizability. Furthermore, the advanced management strategies described may not be available in all health care settings. In calculating the odds ratio for type A dissections, we could not retrieve thoracic aortic diameters for all 2558 TAVR patients due to nonstandardized radiographic protocols, outside hospital studies, and some TAVR cases without preoperative CTA; however, we obtained data for more than 80% of cases, likely reflecting the overall TAVR cohort.

## Conclusions

This analysis indicates that aortic dissection post-TAVR is extremely rare. Conventional aortic dissection management principles should remain, with timely open repair providing excellent outcomes for type A dissections and endovascular approaches for complicated type B dissection cases.

## Conflict of Interest Statement

Dr Fukuhara is a consultant for Medtronic Inc, Edwards Lifesciences, Terumo Aortic, and Artivion. Dr Patel is a consultant for Gore and Medtronic Inc. Dr Deeb is an investigator for Medtronic Inc. All other authors reported no conflicts of interest.

The *Journal* policy requires editors and reviewers to disclose conflicts of interest and to decline handling or reviewing manuscripts for which they may have a conflict of interest. The editors and reviewers of this article have no conflicts of interest.

## Figures and Tables

**Table E1:** Perioperative aortic risk factors in patients experiencing aortic dissections during or after transcatheter aortic valve replacement (TAVR)

Prevalence/incidence	Dilated thoracic aorta	Horizontal aorta	TAVR migration	Bicuspid valve	Genetically triggered aortic disease	Other risk factors
	73.3% (11/15)	46.7% (7/15)	40.0% (6/15)	20.0% (3/12)	0% (0/15)	66.7% (10/15)
Patient no.						
1	Yes	Yes				Previous ascending intramural hematoma
2	Yes	Yes				Abdominal aortic aneurysm
3	Yes					
4	Yes	Yes				Immunosuppression
5	Yes					Penetrating aortic ulcer at STJ
6	Yes		Yes	Yes		
7	Yes	Yes	Yes	Yes		Long and tortuous aorta
8	Yes			Yes		Autograft aneurysm
9	Yes	Yes	Yes			Long and tortuous aorta
10		Yes	Yes			
11	Yes					
12		Yes	Yes			Abdominal aortic aneurysm Penetrating aortic ulcer
13	Yes		Yes			
14						Gothic arch
15						Gothic arch Long and tortuous aorta

Values are presented as % (n/N). *STJ*, Sinotubular junction.
